# Surveillance Program for Diagnosis of HCC in Liver Cirrhosis: Role of Ultrasound Echo Patterns

**DOI:** 10.1155/2017/4932759

**Published:** 2017-05-30

**Authors:** Maurizio Soresi, Antonino Terranova, Anna Licata, Antonietta Serruto, Giuseppe Montalto, Giuseppe Brancatelli, Lydia Giannitrapani

**Affiliations:** ^1^Biomedical Department of Internal Medicine and Specialties (Di.Bi.M.I.S.), University of Palermo, Palermo, Italy; ^2^Section of Radiology, Di.Bi.Me.F., University of Palermo, Palermo, Italy

## Abstract

International guidelines suggest ultrasound surveillance for hepatocellular carcinoma (HCC) early diagnosis in liver cirrhosis (LC) patients, but 40% of nodules* <*2 cm escape detection. We investigated the existence of an ultrasound pattern indicating a higher risk of developing HCC in patients under surveillance. 359 patients with LC (Child-Pugh A-B8) underwent ultrasound screening (median follow-up 54 months, range 12–90 months), liver function tests, alpha-fetoprotein assay, and portal hypertension evaluation. Echo patterns were homogeneous, bright liver, coarse, coarse small nodular pattern, and coarse large nodular pattern. During follow-up 13.9% developed HCC. At multivariate analysis using Cox's model alpha-fetoprotein, coarse large nodular pattern, portal hypertension, and age were independent predictors of HCC development. Kaplan-Meier estimates of HCC cumulative risk in relation to the baseline echo patterns showed risk of 75% in coarse large nodular pattern patients, 23% coarse small nodular pattern, 21% coarse pattern, 0% homogeneous, and bright liver echo patterns (log-rank test = 23.6, *P* < 0.001). Coarse large nodular pattern indicates a major risk factor for HCC as 40.7% of patients with this pattern developed HCC. Homogeneous and bright liver echo patterns and the absence of portal hypertension were not related to HCC. This observation could raise the question of possibly modifying the follow-up timing in this subset of patients.

## 1. Introduction

International guidelines of the screening programs for the early detection of hepatocellular carcinoma (HCC) in cirrhosis patients suggest an ultrasound every six months as the first level of investigation [1–5]. Their main goal is to diagnose the so-called very early HCC, that is, a neoplastic nodule measuring <2 cm [2]. This diagnosis is not always easy, both due to the relatively low sensitivity of the tool, which in very early HCC does not exceed 60% [6, 7], and due to the pattern of presentation at onset, which is sometimes multinodular or infiltrative [8, 9]. Therefore, other indicators to select patients who may have a higher risk of progression in HCC are necessary [10]. Retrospective studies indicate that the* coarse nodular* pattern is a risk factor for the development of HCC [11–15]. Coarse echo pattern is the most common one found in liver cirrhosis (LC) [11]; it is defined coarse nodular by the detection within the liver of small multiple hypoechoic nodular images (<1 cm) at US. A coarse large nodular pattern (CLNP) presents nodules >5 mm, while in a coarse small nodular pattern (CSNP) nodules are <5 mm [11–15]. Histological studies on cirrhotic liver have shown the risk of evolution of these macronodules in HCC [16]. Although the international guidelines recognize the coarse nodular pattern as a risk factor for HCC, they do not recommend a closer follow-up when it is present [2].

In our clinical practice we have anecdotally observed a correlation between coarse nodular pattern and evolution into hepatocellular carcinoma. Consequently, to put this observation in perspective, we carried out a longitudinal study on a cohort of patients with LC prospectively followed from January 2007 to June 2014 in a surveillance program for the early detection of HCC. Our aim was to test the hypothesis that an echo pattern may be associated with a greater or lower risk of evolution to HCC and, in this case, if it is advisable to monitor these patients at shorter or longer follow-up intervals.

## 2. Materials and Methods

### 2.1. Patient Enrolment

We prospectively enrolled consecutive patients with LC of different etiologies, who routinely underwent the surveillance program in accordance with the international guidelines [2, 3]. Exclusion criteria were the following: (a) patients with a history of malignancy; (b) patients with hepatic nodules with suspected malignancy at the first ultrasound; (c) patients of age > 80 years; (d) patients with Child-Pugh class > B 9; and (e) when ultrasound was difficult to perform due to obese habitus or interference from gas in the bowel.

From January 2008 to June 2015, a total of 425 patients were enrolled, but 66 (15.5%) were excluded for reasons above mentioned. 359 patients were thus included in the study (178 M, 181 F), with a mean age of 64.9 ± 9.4 years. The median follow-up was 54 months (12–90 months). Sixty-one of the 359 patients were lost during follow-up due to death or dropout. However, all 61 had a minimum follow-up period of at least 12 months and were therefore also included in the analysis.

The study was carried out under informed consent according to protocols approved by the Biomedical Department of Internal Medicine and Specialties (DIBIMIS) Institutional Review Board (IRB)

A questionnaire designed to assess clinical history, onset of liver disease and its etiology, comorbidities, and medication was administered to all the patients included. All patients also underwent a physical examination, complete blood count, and kidney and liver function tests and were then classified according to the Child-Pugh score [17]. If the etiology of liver disease was unknown, HBsAg, anti-HDV, anti-HCV, and iron serum marker (ferritin, serum iron, and transferrin saturation) assays were performed. Non-organ-specific autoantibodies (ANA, AMA, ASMA, and LKM1) were assayed in patients negative for viral and iron marker screening. Alpha 1 fetoprotein (AFP) assay was performed in all patients every six months.

### 2.2. Abdominal Ultrasound

Ultrasound (US) examinations were performed in the morning after an overnight fast of at least 10 hours, using a 5000 Philips HDI machine with a 2–5 MHz convex probe.

**Figure 1:**
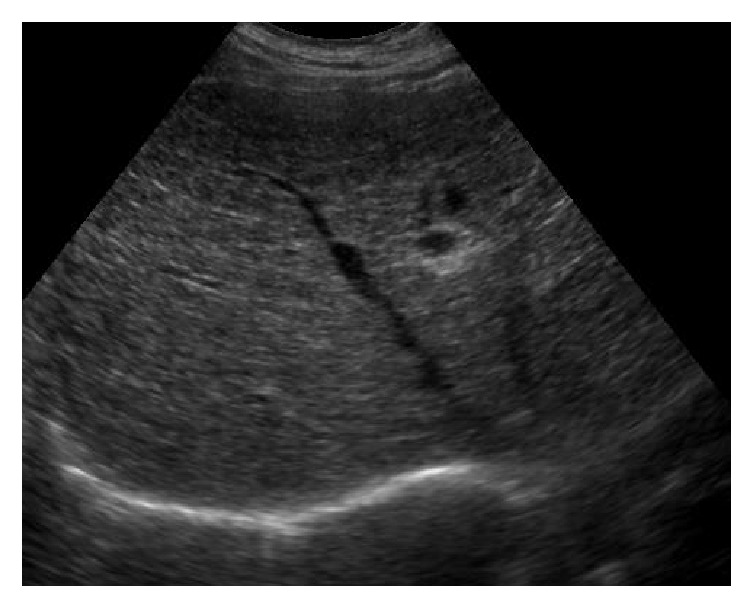
Coarse echo pattern (see text).

**Figure 2:**
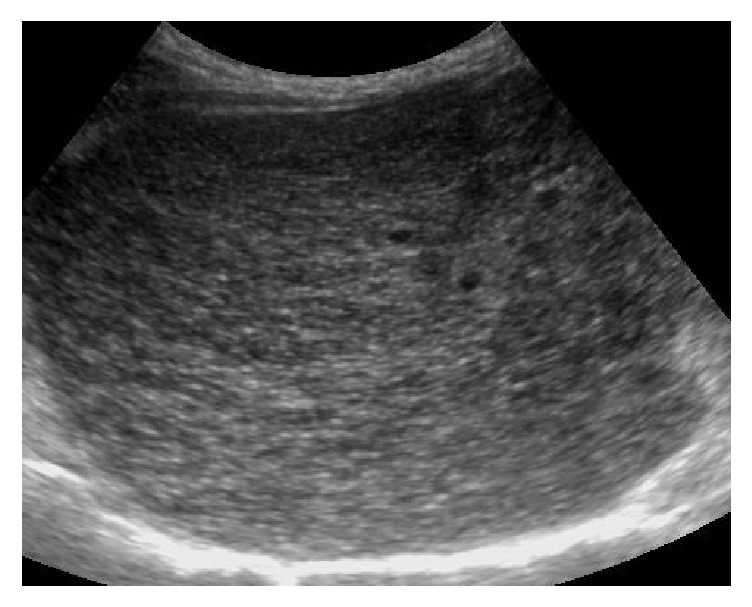
Hypoechoic nodules < 5 mm in diameter on the background of the coarse echo pattern.

**Figure 3:**
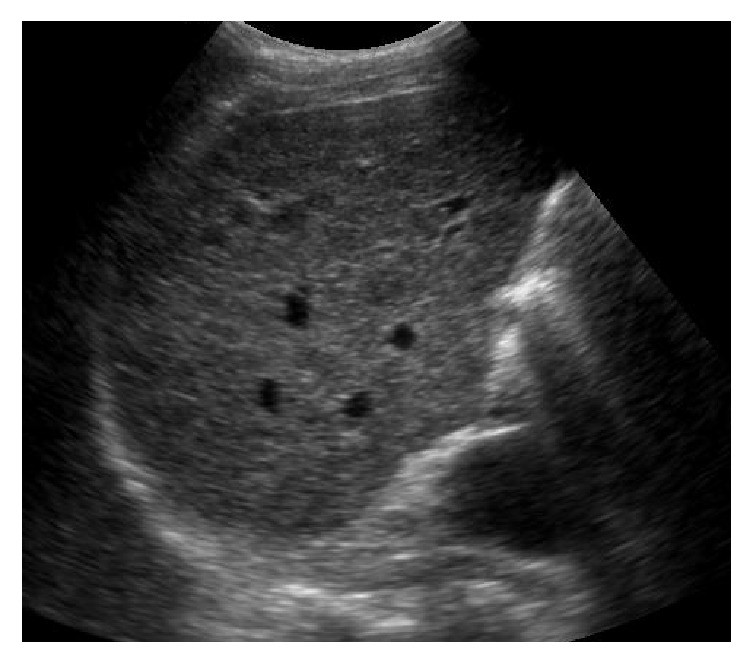
Hypoechoic nodules > 5 mm in diameter on the background of the coarse echo pattern.

Based on the US pattern, patients were divided into five groups:

1. Homogeneous (H): echoes being homogeneously distributed and echogenicity was slightly or not increased.
2. Bright liver (BL): according to the classical definition [18].
3. Coarse pattern (C): characterized by “pinhead” echoes which are coarse and not homogeneously distributed, without posterior beam attenuation and without formation of nodules [18, 19] (Figure 1).
4. Coarse small nodular pattern (CSNP): echo pattern showing scattered hypoechoic nodules up to 3–5 mm in diameter on the background of the coarse echo pattern described above [14] (Figure 2).
5. Coarse large nodular pattern (CLNP): showing scattered hypoechoic nodules > 5 mm in diameter on the background of the coarse echo pattern mentioned above [14] (Figure 3).

Portal vein diameter (PVD), longitudinal diameter of spleen (LDS), and reduction in the respiratory variations of splenic and mesenteric vein diameters were measured in accordance with the literature data and EFSUMB guidelines [20–22]. Normal values were those recommended (reduction of the respiratory variations of splenic and mesenteric vein diameters) by the same guidelines [22].

The platelet to spleen ratio was calculated as described previously by Giannini and colleagues as the ratio between platelet number/mm^3^ and the bipolar diameter of the spleen in millimeters (cut-off 909) [23].

US was performed by two operators (MS, AT) with comparable ability; they had the same professional background, having been trained in this specific field, and both had over a decade of experience.

To reduce interobserver variability of both operators, a set of standard images with H, BL, C, and CSNP was used to assess echo patterns as in Caturelli's work and Kitamura's work figures for the CLNP [11, 14].

Before the study, the ultrasound operators agreed on general roles to follow in the procedure of examination, and they participated in a short training program according to previous ultrasound studies performed in other training [21, 24].

After training, skilled operators identified the possible sources of interobserver variability and issued a strict protocol.

The echo pattern was known to the operators during every serial US examination.

### 2.3. Diagnosis and Follow-Up

LC was diagnosed by histology in 20% of cases; in the remaining cases diagnosis was made on the basis of clinical (presence of spider nevi, palmar erythema, and ascites), endoscopic (esophageal varices or congestive gastropathy), ultrasound (irregular liver surface, hypertrophy of the left segments, ascites, and signs of portal hypertension) parameters [24], and laboratory abnormalities (INR elongation, hypoalbuminemia, increased gamma globulin, and thrombocytopenia). Patients with LC were staged according to the Child-Pugh clinical classification [17].

HCC was diagnosed in accordance with the AASLD guidelines [2, 3] and staged according to the Barcelona Clinic Liver Cancer (BCLC) staging [25].

Patients underwent a medical examination, liver function tests, and AFP assay, as well as ultrasound every six months, with a variability ranging ±1 month in 20% of total examinations.

Nodules showing growth over time or onset of new lesions >1 cm, in accordance with the guidelines, were considered as potential HCC and radiological examinations or biopsy were performed, as set out in the specific guidelines [2, 3].

*Portal Hypertension Diagnosis*. Patients were considered to have portal hypertension if they had

1. endoscopic signs of portal hypertension, that is, presence of esophageal varices, gastric varices portal hypertensive gastropathy, and gastric antral vascular ectasia,
2. ascites and/or collateral circulation,
3. at least 2 of these signs: portal diameter > 1.2 cm, respiratory variations < 40%, and platelet to spleen ratio < 909.

According to the absence/presence of portal hypertension, patients were labeled as 0/1, respectively.

### 2.4. Statistical Analysis

Data were expressed as mean ± SD if the distribution was normal, otherwise as median and range (min–max). Differences between the means of the various groups were calculated by ANOVA. Fisher's exact test, *χ*^2^, and Mantel Haenszel *χ*^2^  (*χ*^2^_MH_), were used when appropriate. Weighted kappa (*k*) statistics were used to evaluate interobserver agreement for echo pattern definition (scored 0/1). The kappa (*k*) value was scored according to Landis and Koch [26]. The strength of concordance was classified as follows: *k* = 0, none; *k* < 0.21, slight; *k* = 0.21–0.4, fair; *k* = 0.41–0.60, moderate; *k* = 0.61–0.8, substantial; *k*≥ 0.81, perfect [26]. To assess which variables measured at baseline were predictive of degeneration to HCC, the univariate Cox proportional hazards model (Hr) was fitted to each variable. All variables with a *P* < 0.05 underwent multivariate analysis to assess their value as independent predictors [27].

The Kaplan-Meier method was used to estimate the risks of HCC degeneration associated with liver echo pattern at enrolment. The log-rank test was used to estimate the probability of cumulative risk of HCC associated with the liver echo pattern [28].

The time of observation used in calculating the risk of HCC began at enrolment and ended when liver cancer was diagnosed, or when the patient died or at the last check-up, whichever came first. The Statistical Software SPSS version 22.0 was used for the statistical analysis. *P* < 0.05 was considered significant.

## 3. Results

### 3.1. Overview of the Cohort

The demographic, clinical, and stage of liver disease data are shown in Table 1. About one-third of patients had Diabetes Mellitus. 316/359 (88%) patients were in Child-Pugh class A and 197 (55%) had endoscopic signs of portal hypertension.

HCV infection was the most frequent etiology, being present in 260 patients (72.3%), followed by HBV in 24 cases (6.7%, of which 1.1% had anti HDV). 35 cases were of cryptogenic etiology (9.7%), which included 7 patients with a history of metabolic syndrome, 17 cases (4.7%) were in the alcohol group, and 15 cases (4.1%) had autoimmune liver diseases (including 2 patients with autoimmune hepatitis, 2 with primary sclerosing cholangitis, and 11 with primary biliary cirrhosis). The mixed/other forms were 9 (2.5%, including 2 with hemochromatosis).

In total, 90 patients (25%) with HCV-associated LC had completed at least one course of antiviral treatment (Peginterferon alone or Peginterferon plus ribavirin), while all patients with HBV-associated LC were on treatment with nucleoside/nucleotide analogs.

### 3.2. Distribution of Echo Patterns

Overall, for the various echo patterns, *k* was 0.85 (95% CI 0.75–0.9), that is, perfect agreement according to Landis' score.  Table 2 shows the *k* concordance for each single echo pattern, which oscillated between substantial and perfect agreement. No discordance was observed for the H pattern.

Table 3 shows the echo patterns at enrolment and the follow-up period of each pattern. There were no significant statistical differences among them (*F* = 0.9; *P* = ns).

**Figure 4:**
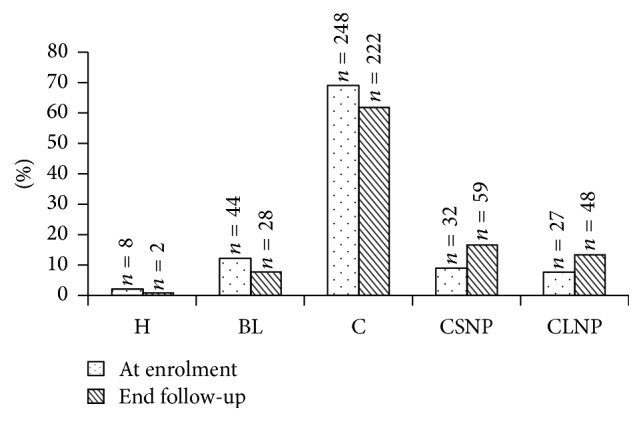
Changes in echo pattern at enrolment and end of follow-up (H, homogeneous; BL, bright liver; C, coarse pattern; CSNP, coarse small nodular pattern; CLNP, coarse large nodular pattern) (*χ*^2^_MH_ = 114,7; *P* = 0.0001).

In 90 subjects (25%) the echo structure changed during the follow-up period.  Figure 4 shows these changes and their distribution at baseline and at the end of the follow-up period. At the end of follow-up the nodular echo patterns (both CSNP and CLNP) had increased in a statistically significant way (*χ*^2^_MH_ = 114,7; *P* = 0.0001). In fifty patients (13.9%; CI 95% 10.5–17.9) LC evolved into HCC during follow-up.

### 3.3. Prognostic Indicators of HCC Evolution According to the Different Echo Patterns and PH

Using the Cox model (Table 4), at univariate analysis many factors were associated at baseline with the evolution in HCC, while at multivariate analysis only AFP: Hr = 1.1 (CI 95%: 1.05–1.2) (*P* < 0.02), CLNP: Hr = 3.4 (CI 95% = 1.6–6.6) (*P* = 0.02), age: Hr = 1.05 (CI 95% 1.02–1.1) (*P* = 0.03), and PH: Hr 2.1 (CI 95%: 1.1–4.1) *P* = 0.03 were found to be independent predictors of HCC. Even when we eliminated AFP from the multivariate model, CLNP, age, and PH were still associated factors of HCC degeneration (data not shown).

The median follow-up time of patients with PH was 49 (12–90) months; in those without PH it was 48 (12–90) months (*P* = ns).

**Figure 5:**
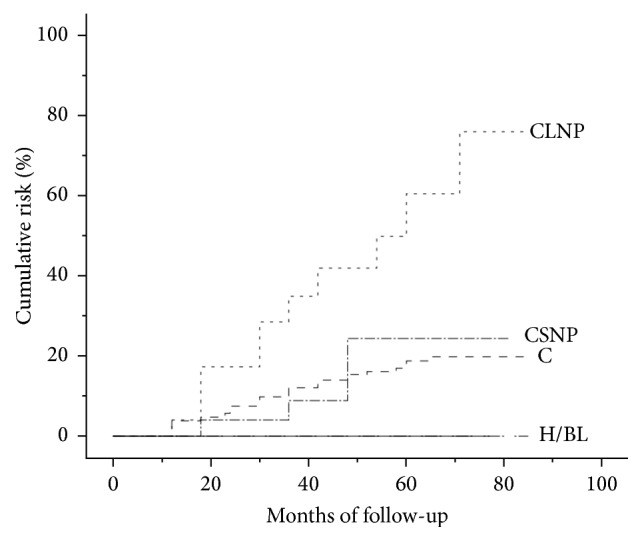
Cumulative risk for echo patterns: H/BL (homogeneous/bright liver), C (coarse), CSNP (coarse small nodular pattern), and CLNP (coarse large nodular pattern). A log-rank test showed significant differences (log-rank test = 23.6, *P* < 0.001).

Figure 5 shows the cumulative risk curves for the development of HCC in relation to the baseline echo pattern. Using the Kaplan-Meier method, the US pattern at the end of follow-up showed a cumulative risk % (±SE) for HCC of 75% (±10%) for patients with CLNP, 23% (±10%) with CSNP, 21% (±3%) with C pattern, and 0% with the H and BL patterns. The log-rank test of the five curves showed a statistically significant difference (log-rank test = 23.6, *P* < 0.001).

Table 5 shows the echo pattern distribution at enrolment in relation to the BCLC Stage. There was no statistically significant association between BCLC Stage and echo patterns at enrolment.

During follow-up, patients who developed more frequently HCC were those with CLNP pattern at enrolment 11/27 (40%; CI 95% 24.4–59.4), in a statistically different manner versus C 35/248 (14%; CI 95% 10.3–14.1) (*P* < 0.002) and versus CSNP 4/32 (12.5%; CI 95% 5.1–28.2) (*P* < 0.0001).

### 3.4. Reliability of Ultrasound

Ultrasound missed 12 nodules, detected by CT or MR, 8/11 were smaller than 2 cm, and 3 were <3 cm. In 1 case the nodule was not detected by ultrasound and suspected because there was an abrupt increase of AFP from 30 to 210 ng/mL without increase in serum levels of AST/ALT; CT confirmed the presence of HCC 2.3 cm. The positive predictive value of ultrasound was 79% (CI 95%; 67–88); the negative predictive value was 96% (CI 95% 93–98%).

## 4. Discussion

Hepatocellular carcinoma is one of the most frequent cancers in the world, with a high mortality rate. Since the main associated risk factor is LC [29], cirrhotic patients undergo six-monthly surveillance programs with ultrasound, aimed at establishing an early diagnosis, which is associated with a greater effectiveness of treatment [1–5].

Unfortunately, tumors > 2 cm are often found, even in patients under surveillance. Early diagnosis is not easy, due to the limited sensitivity of US, not exceeding 60% in very early HCC [6, 7], and to the pattern of tumor spread, which can sometimes be multinodular or infiltrating [8, 9]. The positive and negative predictive values are consistent with data reported in the literature when, as in our study, the gold standard consists of radiological investigations such as CT and MR. The reliability of ultrasound is lower when the gold standard is the histological study of explanted livers [30]. Moreover, not all patients with liver cirrhosis have an equal risk of developing HCC; therefore an increasing number of studies are being targeted to select “at risk” subpopulations to better focus the surveillance programs and reduce costs [10]. Reducing the follow-up interval to three months has not been very useful because this increased the number of false positives (regenerative nodules) and increased costs, without improving the diagnosis rates of very early HCC [31]. In the literature, the coarse nodular pattern has been proposed as an independent risk factor for the onset of hepatocellular carcinoma [11–15]. However, all the studies conducted so far have the limitation of being retrospective and performed with older generation ultrasound equipment.

In our study, the *k* value, using Landis's score, ranged between 0.79 and 1, which suggests that ultrasound has a good degree of reproducibility in defining the different echo patterns of liver cirrhosis, when it is performed by expert operators with specific training using up-to-date equipment as already demonstrated in previous US studies [21, 24] and according to what is recommended by current guidelines [1].

We conducted a longitudinal prospective study, the first to our knowledge, in which we followed a cohort of 359 patients with LC for a mean follow-up period of 54 months (12–90 months). In fifty of these subjects to date LC has evolved into hepatocellular carcinoma. This percentage (13.9%) is in agreement with findings in the current literature [31, 32]. The echo pattern most frequently associated with the neoplastic evolution was the CLNP 11/27 (40%). Using the Cox regression model at multivariate analysis the variables considered as risk factors for the onset of HCC were AFP, the CLNP, and age. Our data, therefore, although limited by the small number of CLNP patients confirm that this pattern has an increased risk for neoplastic degeneration. Moreover, histological studies have found in these subjects an increase in the hepatocellular proliferation index, evaluated with bromouridine [13], with techniques of immunoreactivity for the DNA polymerase-*α* [14] and with the nucleolar organizer regions [15].

It is well known that hepatocarcinogenesis in cirrhosis follows a “multiple steps” model, with the transition from a regenerative nodule, then a dysplastic nodule, and finally HCC [33]. A macronodular liver is probably at a greater risk because this mechanism is activated and can potentially be achieved in a number of different areas. Furthermore, the cirrhotic liver tends to become nodular over time, as confirmed in our study by the statistically significant trend increase (Table 2) in the nodular pattern during the surveillance period, and the pattern that increases most is the macronodular one. We used the Kaplan-Meier curves to estimate the cumulative risk of developing HCC. As shown in Figure 2, the coarse large nodular pattern appears to be significantly more at risk than the other echo patterns. In detail, at the end of follow-up, the risk of developing hepatocellular carcinoma was 75% for the CLNP, 23% for CSNP, and 21% for C.

Portal hypertension has been reported to be associated with a higher risk of HCC in patients with compensated cirrhosis [34]. It is well known, however, that endoscopic signs are specific but poorly sensitive for identifying which patients already have portal hypertension. Recent data suggest that noninvasive parameters can reliably indicate the presence or absence of clinically significant portal hypertension in patients with compensated cirrhosis [35]. In our study we used other noninvasive parameters of portal hypertension included in the guidelines, such as portal vein diameter and respiratory changes, and those already known in the literature, such as the spleen/platelet ratio [20–23]. With these we found that PH was an independent risk factor of neoplastic degeneration. We are aware that these data need to be confirmed, as a major limitation of our study is the lack of HVPG measurements. However, our results are supported by the study of Ripoll, who found that HVPG values > 10 mm Hg, together with low albumin levels and viral etiology, are indicative of neoplastic degeneration in LC patients. Although it is difficult to explain the reasons for such an association, some metabolic pathways of the cirrhotic patient may possibly stimulate portal hypertension and hepatocarcinogenesis, as suggested by the recent finding of heat shock protein increase in portal hypertension [36].

In this study the AFP was also confirmed as an independent risk factor for the development of hepatocellular carcinoma. However, the meta-analysis by Singal et al. has clarified its true role. This marker is a risk factor for HCC, but its evaluation is not very useful because it only slightly enhances US sensitivity in diagnosing early cancer from 64% to 70%, while increasing the cost [7].

When we compared the relationship between the echo patterns at enrolment and the BCLC staging of HCC, we found no statistical association. This result is important as it provides two suggestions: the first is that although the macronodular pattern does indicate a risk of neoplastic transformation, the six-monthly follow-up proposed by the guidelines allows a timely diagnosis of the disease; the second is that the biological aggressiveness of the tumor has probably no relationship with the US pattern and the presence of multiple nodules, as in the CLNP or CSNP, is therefore not predictive for a multifocal evolution.

Finally, similarly to the study by Caturelli et al. [11] none of the HCC cases developed on BL.

## 5. Conclusions

In summary, in this study we found that the CLNP and PH age and AFP are the most significant risk factors for malignant degeneration. While the CLNP group include a small number of patients, the absence of a relationship between the US findings at enrolment and tumor prognosis assessed by the BCLC classification suggests that to obtain an early diagnosis of HCC in the presence of a CLNP it is not necessary to shorten the six-month follow-up interval. In fact the level of risk determines whether to provide surveillance or not while the surveillance interval depends on the rate of tumor growth and the minimum size of tumor at diagnosis consistent with a high cure rate. There is no evidence, so far, data suggesting that higher risk equals more rapid growth. This is important because these patients, due to the lack of liver homogeneity observed at ultrasound, often arouse alarm requiring frequent and repeated imaging examinations, thus increasing the cost of the surveillance programs. However, its association with PH opens the door to new prospects, and further studies are required with histological or molecular marker analyses to allow the selection of higher risk categories. In this case the question could be raised as to whether it would be appropriate to change the follow-up timing in a given subpopulation of patients.

Finally, we are aware that the limited number of patients included in our study has not the power to modify the current timing of US in LC patients; however, they point to implement other studies with a greater number of patients in order to evaluate the opportunity to modify the current timing of US and, at the same time, reduce costs.

## Figures and Tables

**Table 1:** Clinical and laboratory features of the study patients.

	*N* = 359
Age (years)	64.9 ± 9.4
Sex (M/F)	181/178
AST (IU/L)	53 (8–477)
ALT (IU/L)	56 (12–443)
ALB (g/dL)	3.9 ± 0.6
Platelets n/mm^3^	130.000
	(26.000–400.000)
Longitudinal diameter of spleen (mm)	132 ± 26
AFP (ng/mL)	5.2 (0.2–258)
Diabetes Mellitus	119 (33%)
Antiviral treatment	118 (33%)
Child-Pugh Score:	
A5-6	316 (88%)
B7-8	43 (12%)
Endoscopic portal hypertension	197 (55%)
Portal hypertension	237 (66%)

AST, aspartate transaminase; ALT, alanine transaminase; ALB, albumin; AFP, alpha-fetoprotein

Portal hypertension (endoscopic + noninvasive).

**Table 2:** Correlation coefficient (*k*) of the two sonographers for single echo pattern classified according to Landis' score.

	*k* concordance	95% CI	Landis' score
H	1	—	Perfect agreement
BL	0.85	0.80–0.90	Perfect agreement
C	0.88	0.81–0.95	Perfect agreement
CSNP	0.79	0.75–0.83	Substantial agreement
CLNP	0.78	0.73–0.93	Substantial agreement

H, homogeneous; BL, bright liver; C, coarse pattern; CSNP, coarse small nodular pattern; CLNP, coarse large nodular pattern.

**Table 3:** Echo patterns at enrolment and duration of follow-up.

	*n* =	% (IC 95%)	Follow-up in month^*∗*^
H	8	2.3 (1.14–4.3)	48.0 ± 20.6
BL	44	12.2 (IC 95% 9.2–16.0)	48.5 ± 22.1
C	248	69.1 (IC 95% 64.1–73.6)	49.71 ± 23.4
CSNP	32	8.9%; (IC 95% 6.4–12.3)	44.9 ± 22.9
CLNP	27	7.5 (IC 95% 5.2–10.7)	44.5 ± 20.7

^*∗*^(*F* = 0.9; *P* = ns).

H, homogeneous; BL, bright liver; C, coarse pattern; CSNP, coarse small nodular pattern; CLNP, coarse large nodular pattern.

**Table 4:** Risk factors for progression to hepatocellular carcinoma according to Cox's model at univariate and multivariate analysis.

	HR univariate	95% CI	*P*<	HR multivariate	95% CI	*P*<
Age	1.05	1.02–1.08	0.02	1.05	1.02–1.1	0.03
Sex	1.14	0.6–1.9	ns	—	—	
HCV	2.06	0.9–4.5	ns	—	—	
HBV	1.36	0.5–3.8	ns	—	—	
Alcohol	0.43	0.1–3.1	ns	—	—	
Cryptogenetic	0.4	0.1–2.7	ns	—	—	
Autoimmune liver diseases	0.047	0.02–37.1	ns	—	—	
Metabolic	0.8	0.22–111.7	ns	—	—	
H	—	—	—	—	—	
BL	—	—	—	—	—	
C	1.02	0.55–1.90	ns	—	—	
CSNP	1.02	0.36–2.84	ns	—	—	
CLNP	3.84	1.9–7.51	0.02	3.4	1.6–6.6	0.01
AFP ng/ml	1.1	1.06–1.2	0.0001	1.1	1.05–1.2	0.02
AST IU/L	1.04	1.01–1.07	0.03	—	—	—
ALT IU/L	1.1	1.03–1.2	0.0001	—	—	—
ALB g/dl	0.51	0.31–0.81	0.005	—	—	—
Antiviral treatment	0.9	0.7–2	ns	—	—	—
Child-Pugh score (A5-B8)	0.9	0.65–1.21	ns	—	—	—
Diabetes Mellitus	1.12	0.8–2.15	ns	—	—	
Endoscopic portal hypertension	1.78	0.75–4.25	ns	—	—	—
Portal hypertension	2.3	1.18–4.5	0.02	2.1	1.1–4.1	0.03

HR, Hazard Ratio; CI, Confidence Interval; H, homogeneous; BL, bright liver; C, coarse pattern; CSNP, coarse small nodular pattern; CLNP, coarse large nodular pattern; AFP, alpha-fetoprotein; AST, aspartate transaminase; ALT, alanine transaminase; ALB, albumin; portal hypertension (endoscopic + noninvasive).

**Table 5:** Distribution of HCC and BCLC staging in relation to the echo patterns at enrolment.

Echo pattern at enrolment	H	BL	C	CSNP	CLNP	
	*n* = 8	*n* = 44	*n* = 248	*n* = 32	*n* = 27	
HCC *n* = 50	*n* = 0	*n* = 0	*n* = 35	*n* = 4	*n* = 11	

BCLC Stage						
0	0	0	15	1	6	
A	0	0	17	3	5	
B	0	0	2	0	0	
C	0	0	1	0	0	
D	0	0	0	0	0	*χ* ^2^ = 3.5; *P* = NS

H, homogeneous; BL, bright liver; C, coarse pattern; CSNP, coarse small nodular pattern; CLNP, coarse large nodular pattern; HCC, hepatocellular carcinoma; BCLC, Barcelona Clinic Liver Cancer.

## References

[1] Bruix J., Sherman M., Llovet J. M. (2001). Clinical management of hepatocellular carcinoma, conclusions of the barcelona-2000 EASL conference. *Journal of Hepatology*.

[2] Bruix J., Sherman M., Practice Guidelines Committee, American Association for the Study of Liver Diseases (2005). Management of hepatocellular carcinoma. *Hepatology*.

[3] Bruix J., Sherman M. (2011). American Association for the Study of Liver Diseases. Management of hepatocellular carcinoma: an update. *Hepatology*.

[4] European association for the Study of the Liver (2012). EASL-EORTC clinical practice guidelines: management of hepatocellular carcinoma. *Journal of Hepatology*.

[5] Italian Association for the Study of the Liver (AISF), AISF Expert Panel, AISF Coordinating Committee, Bolondi L., Cillo U., Colombo M. (2013). Position paper of the Italian Association for the Study of the Liver (AISF): the multidisciplinary clinical approach to hepatocellular carcinoma. *Digestive and Liver Diseases*.

[6] Bolondi L. (2003). Screening for hepatocellular carcinoma in cirrhosis. *Journal of Hepatology*.

[7] Singal A., Volk M. L., Waljee A., Salgia R., Higgins P., Rogers M. A. (2009). Meta-analysis: surveillance with ultrasound for early-stage hepatocellular carcinoma in patients with cirrhosis. *Alimentary Pharmacology and Therapeutics*.

[8] Stroffolini T., Andreone P., Andriulli A. (1998). Characteristics of hepatocellular carcinoma in Italy. *Journal of Hepatology*.

[9] Soresi M., La Spada E., Giannitrapani L., Campagna E., Di Gesaro V., Granà W. (2010). Hepatocellular carcinoma: comparison of two different periods at the same center. *European Journal of Internal Medicine*.

[10] Sherman M., Klein A. (2004). AASLD single-topic research conference on hepatocellular carcinoma: conference proceedings. *Hepatology*.

[11] Caturelli E., Castellano L., Fusilli S. (2003). Coarse nodular US pattern in hepatic cirrhosis: risk for hepatocellular carcinoma. *Radiology*.

[12] Mikami N., Ebara M., Yoshikawa M., Ohto M. (1990). Relationship between ultrasound-findings of low-echoic nodule of hepatic parenchyma in liver cirrhosis and development of hepatocellular carcinoma. *Japanese Journal of Gastroenterology*.

[13] Tarao K., Hoshino H., Shimizu A. (1995). Patients with ultrasonic coarse-nodular cirrhosis who are anti-hepatitis C virus-positive are at high risk for hepatocellular carcinoma. *Cancer*.

[14] Kitamura S., Iishi H., Tatsuta M. (1995). Liver with hypoechoic nodular pattern as a risk factor for hepatocellular carcinoma. *Gastroenterology*.

[15] Azzaroli F., Colecchia A., Lodato F. (2006). A statistical model predicting high hepatocyte proliferation index and the risk of developing hepatocellular carcinoma in patients with hepatitis C virus-related cirrhosis. *Aliment Pharmacol Ther*.

[16] Hytiroglou P., Theise N. D., Schwartz M., Mor E., Miller C., Thung S. N. (1995). Macroregenerative nodules in a series of adult cirrhotic liver explants: issues of classification and nomenclature. *Hepatology*.

[17] Pugh R. N. H., Murray Lyon I. M., Dawson J. L. (1973). Transection of the oesophagus for bleeding oesophageal varices. *The British Journal of Surgery*.

[18] Joseph A. E. A., Saverymuttu S. H., Al-Sam S., Cook M. G., Maxwell J. D. (1991). Comparison of liver histology with ultrasonography in assessing diffuse parenchymal liver disease. *Clinical Radiology*.

[19] Soresi M., Giannitrapani L., Florena A. M. (2009). Reliability of the bright liver echo pattern in diagnosing steatosis in patients with cryptogenic and HCV-related hypertransaminasaemia. *Clinical Radiology*.

[20] Bolondi L., Gandolfi L., Arienti V. (1982). Ultrasonography in the diagnosis of portal hypertension: diminished response of portal vessels to respiration. *Radiology*.

[21] Sabbá C., Merkel C., Zoli M. (1995). Interobserver and interequipment variability of echo-doppler examination of the portal vein: effect of a cooperative training program. *Hepatology*.

[22] Berzigotti A., Piscaglia F. (2011). Ultrasound in portal hypertension--part 1. *Ultraschall in der Medizin*.

[23] Giannini E., Botta F., Borro P. (2003). Platelet count/spleen diameter ratio: proposal and validation of a non-invasive parameter to predict the presence of oesophageal varices in patients with liver cirrhosis. *Gut*.

[24] Soresi M., Noto D., Cefalù A. B. (2013). Nonalcoholic fatty liver and metabolic syndrome in Italy: results from a multicentric study of the Italian Arteriosclerosis society. *Acta Diabetologica*.

[25] Forner A., Reig M. E., de Lope C. R., Bruix J. (2010). Current strategy for staging and treatment: the BCLC update and future prospects. *Seminars in Liver Disease*.

[26] Landis J. R., Koch G. G. (1977). The measurement of observer agreement for categorical data. *Biometrics*.

[27] Cox D. R. (1972). Regression models and life-tables. *Journal of the Royal Statistical Society*.

[28] Kaplan E. L., Meier P. (1958). Nonparametric estimation from incomplete observations. *Journal of the American Statistical Association*.

[29] Fattovich G., Stroffolini T., Zagni I., Donato F. (2004). Hepatocellular carcinoma in cirrhosis: incidence and risk factors. *Gastroenterology*.

[30] Hanna R. F., Miloushev V. Z., Tang A. (2016). Comparative 13-year meta-analysis of the sensitivity and positive predictive value of ultrasound, CT, and MRI for detecting hepatocellular carcinoma. *Abdominal Radiology*.

[31] Trinchet J.-C., Chaffaut C., Bourcier V. (2011). Ultrasonographic surveillance of hepatocellular carcinoma in cirrhosis: A randomized trial comparing 3- and 6-month periodicities. *Hepatology*.

[32] El-Serag H. B. (2012). Epidemiology of viral hepatitis and hepatocellular carcinoma. *Gastroenterology*.

[33] Choi J.-Y., Lee J.-M., Sirlin C. B. (2014). CT and MR imaging diagnosis and staging of hepatocellular carcinoma: part I. Development, growth, and spread: key pathologic and imaging aspects. *Radiology*.

[34] Ripoll C., Groszmann R. J., Garcia-Tsao G. (2009). Hepatic venous pressure gradient predicts development of hepatocellular carcinoma independently of severity of cirrhosis. *Journal of Hepatology*.

[35] Berzigotti A., Seijo S., Arena U. (2013). Elastography, spleen size, and platelet count identify portal hypertension in patients with compensated cirrhosis. *Gastroenterology*.

[36] Buck M., Garcia-Tsao G., Groszmann R. J. (2014). Novel inflammatory biomarkers of portal pressure in compensated cirrhosis patients. *Hepatology*.

